# Rats (*Rattus norvegicus*) flexibly retrieve objects’ non-spatial and spatial information from their visuospatial working memory: effects of integrated and separate processing of these features in a missing-object recognition task

**DOI:** 10.1007/s10071-015-0915-8

**Published:** 2015-08-27

**Authors:** Corrine Keshen, Jerome Cohen

**Affiliations:** Department of Biological Sciences, University of Windsor, Windsor, ON N9B 3P4 Canada; Early Childhood Multidisciplinary Intervention, 699 Eglinton Avenue West #202, Toronto, ON M5N 1C6 Canada; Department of Psychology, University of Windsor, Windsor, ON N9B 2P4 Canada

**Keywords:** Rat spatial cognition, Working memory, Object recognition

## Abstract

After being trained to find a previous missing object within an array of four different objects, rats received occasional probe trials with such test arrays rotated from that of their respective three-object study arrays. Only animals exposed to each object’s non-spatial features consistently paired with both its spatial features (feeder’s relative orientation and direction) in the first experiment or with only feeder’s relative orientation in the second experiment (Fixed Configuration groups) were adversely affected by probe trial test array rotations. This effect, however, was less persistent for this group in the second experiment but re-emerged when objects’ non-spatial features were later rendered uninformative. Animals that had both types of each object’s features randomly paired over trials but not between a trial’s study and test array (Varied Configuration groups) were not adversely affected on probe trials but improved their missing-object recognition in the first experiment. These findings suggest that the Fixed Configuration groups had integrated each object’s non-spatial with both (in Experiment 1) or one (in Experiment 2) of its spatial features to construct a single representation that they could not easily compare to any object in a rotated probe test array. The Varied Configuration groups must maintain separate representations of each object’s features to solve this task. This prevented them from exhibiting such adverse effects on rotated probe trial test arrays but enhanced the rats’ missing-object recognition in the first experiment. We discussed how rats’ flexible use (retrieval) of encoded information from their visuospatial working memory corresponds to that of humans’ visuospatial memory in object change detection and complex object recognition tasks. We also discussed how foraging-specific factors may have influenced each group’s performance in this task.

**Electronic supplementary material:**

The online version of this article (doi:10.1007/s10071-015-0915-8) contains supplementary material, which is available to authorized users.

## Introduction

A major challenge in comparative cognition is to devise memory tasks for animals that yield similar effects as those used in research with humans (Thorpe et al. 2004). For example, human visuospatial working memory as measured by change detection accuracy for briefly presented (up to 3000 ms) arrays of two-dimensional items is limited to four items (Eng et al. 2005; Luck and Vogel 1997; Pashler 1988; Riggs et al. 2006). After viewing a study array of items, subjects are presented a test array of a subset of these items and must indicate whether any of them differ in one or more features (e.g., color, location, shape, orientation) from those of the original items. Subjects’ accuracy for detecting such changes typically declines as the number of the ‘study’ array items (its set size) increases from four to twelve items. Although a comparable change detection task developed for pigeons (Wright et al. 2010) and monkeys (Elmore et al. 2011) produces similar set size effects, this type of preparation is inappropriate for the less visually adept rat. Instead, attempts to determine capacity limits in rats’ working memory by increasing the number of arms in the radial maze (Cole and Chapelle-Stephenson 2003) or of objects in a reinforced version of a novel object recognition task (Cohen et al. 2010) have not produced similar effects. In the more recent study, rats first had to find food (sunflower seeds) beneath several ‘junk’ objects within a ‘study’ array in a large foraging arena and then, after a brief 2-min interval, had to find food beneath a new object or a previously visited one moved to a new location within the test array. Increasing the study array’s set size from 3 to 12 objects actually improved rats’ performance for locating the baited target object(s).

Despite a failure to establish reliable limits in rats’ visuospatial working memory in that new or changed object recognition task, Arain et al. (2012) presented evidence for limited capacity in rats’ visuospatial working memory in a missing-object recognition task. In that study, rats received a total of four different or identical adjacent objects arranged in a square array. In any trial, rats were exposed to any three of these objects in the ‘study’ array from which they retrieved sunflower seeds and then were exposed to all four objects in the ‘test’ array where they could only retrieve a reward beneath the previously missing (target) object. During initial training, the location of the study and test arrays and the positions of the different objects varied over trials but not between any trial’s study and test array. On some post-acquisition, probe trials, however, a trial’s test array’s location within the larger foraging chamber or the position of a previously absent object had been changed from that of its study array. Under these conditions, rats showed better missing-object recognition accuracy than on regular baseline trials. This enhancement effect suggests that during training, rats represented each object as consisting of separate local, global spatial positions, and non-spatial features. On occasional probe trials where each object’s spatial features had been changed on its test array, rats were able to reduce the amount of spatial information they needed to retrieve from working memory to find the missing object.

If rats can adjust the amount of separately encoded spatial information they need to retrieve about objects during foraging, one would expect that humans could do the same in tests of their visuospatial memory. In a series of experiments with multi-featured items (Vogel et al. 2001, Experiments 11–15), subjects’ change detection accuracy as a function of study set size was similar whether they were instructed to remember either only one type of feature of each item (e.g., color or orientation of rectangles; color of internal smaller or larger external square of each object) or both features, the conjunction condition. These findings seemed to indicate that subjects could not help but integrate both features of each object and retrieve these integrated representations despite encoding instructions (object-integration hypothesis). Only when each object consisted of two differently colored small and large squares diagonally adjacent to each other, did subjects display superior change detection when instructed to retain the colors of only squares of a specific size than of both sizes (Vogel et al. 2001, Experiment 16). Except for that experiment, subjects were generally unable to adjust the amount of information from pre-trial instructions that they only needed to separately encode and retrieve. The object-feature integration hypothesis predicts that increasing set size should also similarly reduce change detection accuracy in uni-featured and multi-featured items. However, other studies (Wheeler and Treisman 2002, Experiments 1 and 2; Alvarez and Cavanagh 2004; Eng et al. 2005), reveal that subjects show greater declines for multi-featured than for uni-featured objects (e.g., small colored squares inside larger differently colored squares versus only small or large colored squares; differently oriented shaded cubes or faces versus colored squares or polygons). Of particular interest to us were the findings from studies where subjects received only multi-featured items but were told to determine whether a trial’s test array differed from that of the study array because either one type of feature might have been changed on two items (color or location of squares: Wheeler and Treisman 2002, Experiment 3A; color or geometric shape: Treisman and Zhang 2006) or had only been switched between two items. Subjects were better at detecting a change for single features whether or not they were told which type of feature might be changed than for detecting a change in relationship between unchanged features (their binding). These findings suggest that subjects do not automatically integrate each item’s features after a single exposure of an array of items. Later research (Sala and Courtney 2009) did show that pre-trial cueing biased subjects’ attention to items’ non-spatial or spatial features without affecting their retrieval of either type of information among a small set of three items. Prior to being tested to determine whether a test object matched any of the study objects based on pre-trial cueing instructions, subjects had to respond on a perceptual discrimination task on letters and numbers superimposed on two fractal-patterned squares, one of which was previously presented in the three-item study array. Subjects responded faster to the correct superimposed number (target) when it occurred on a square containing the spatial or non-spatial feature they had been previously cued might change in the subsequent test array than when it occurred on the square with a different (non-cued) combination of these features.

Pre-trial instructions might direct a subject’s attention to one type of feature over another or to both in each object. But such differences in attention do not immediately produce different types of representations of a multi-featured item, one consisting of its feature-specific representation and the other of a representation of integrated features. Rather subjects may have to expend more concerted effort in learning to distinguish among multi-featured objects to form a distinct unitary representation of each object. The most striking evidence for this proposition occurs from another series of studies on recognition of natural multi-featured objects such as human faces, dogs, houses, and automobiles (Diamond and Carey 1986; Tanaka and Sengco 1997; Sangrigoli and de Schonen 2004; Curby et al. 2009) or of nonsense objects, ‘greebles’ (Gauthier and Tarr 1997; Gauthier et al. 2000). Taken together, the findings from these studies reveal that subjects having become experts at identification of specific objects within a category are less accurate in recognizing either inverted than upright objects within that category or in detecting if the changed spatial or non-spatial characteristics of features (e.g., eyes, nose, mouth) within such normally oriented objects have been changed than are ‘novices.’ Research with other primates also reveals that chimpanzees show the object-inversion effect for faces of unfamiliar conspecifics but not for those of humans or capuchin monkeys or for automobiles (Parr et al. 1998) or houses (Parr and Heintz 2006), but rhesus monkeys show it for both conspecific and chimpanzee faces but not for houses until after being extensively trained with upright exemplars (Parr and Heintz 2008). Gauthier and Logothetis (2000) maintain that highly similar behavioral and brain functioning for complex natural and artificial object recognition exists between monkeys and humans. According to Maurer et al. (2002), humans and some other primates integrate the internal features of such complex objects into holistic representations after extensive exposure to such complex objects’ in their ‘normal’ upright orientations. Thus, the inversion effect reflects the inability of ‘experts’ to use such holistic representations of upright objects as comparisons with reoriented objects. Based on these findings, we would expect that subjects in the change detection studies already cited could have developed unitary representations with simpler multi-featured items (e.g., color patches at different locations) if they had been repeatedly exposed to a given set of these objects to prevent them from retrieving one feature without the other in each item despite any pre-trial cueing.

The present study examined whether conditions that seemingly promote separate or combined processing of different features within multi-featured objects by humans in change detection or object recognition tasks also operate for rats in a missing-object recognition foraging task. Our study was an extension of research by Arain et al. (2012) that considered rats to have separately processed each object’s non-spatial and spatial features. In our study, we asked whether some rats would integrate each object’s spatial and non-spatial features into a unitary representation when its features had varied neither over nor within trials, that is, between their study and test arrays during training (the Fixed Array Configuration group). Conversely, we considered that other rats would only be able to separately represent each object’s spatial and non-spatial features that had been varied over trials but not within any trial during training (the Varied Array Configuration group) as in Arain et al. (2012). If each group of rats had developed a different way of processing objects’ spatial and non-spatial information, they should react differently whenever they later encountered a trial’s test array with one or the other type of features having been made irrelevant or uninformative. We expected that rats trained with fixed configurations would be adversely affected by being unable to match a representation of a missing object’s integrated spatial and non-spatial features with any of the objects in a changed test array. Rats trained with varied array configurations would be enhanced rather than disrupted by being able to retrieve only that separately encoded information relevant for finding a remaining baited food site (feeder). A subsidiary but related question concerns whether rats trained to form unitary representations of each object might also form a holistic representation of the overall shape of the array of these objects when the orientation of their study and test arrays remained unchanged than when it was changed over trials. This question was prompted by previously cited research showing that the adverse complex object-inversion effect was a function of amount of discrimination training with multi-featured objects in ‘upright’ orientations. According to the array-shape hypothesis, only rats previously trained with unaltered oriented arrays would be adversely affected for finding a missing object in an altered oriented test array. We ran two experiments to test these predictions. In the first experiment, rats in each group were trained with unaltered oriented arrays over trials, while in a second experiment, other rats in each group were trained with altered oriented arrays. According to the array-shape orientation hypothesis, within-trial changes in test array orientation would only disrupt Fixed Configuration group’s rats’ accuracy for fining a missing object in the first experiment but should enhance it for the Varied Configuration group’s rats in either experiment.

Although the focus of this study was on the effects of test array manipulations on post-acquisition performance in each group, we also expected rats in the Fixed Configuration group to more easily acquire the missing-object recognition task than rats in Varied Configuration group. This prediction follows from the notion that by integrating spatial and non-spatial features of each object, rats in the Fixed Configuration group should require less information than those in the Varied Information group to find a missing object on training trials.

## Methods

### Animals

A total of 24 male Long–Evans hooded rats from Charles River Breeding Farms, St. Constant, Quebec, served in this study. Half these animals served in the first experiment, and other half served in the second experiment. All animals were between 3 and 5 months old and weighed between 300 and 400 g at the beginning of each experiment. They were fed 20–25 g of food (Purina Rodent Chow) for 2 h in individual holding cages following each experimental session and before being returned to their large group cages (three rats per cage) in our colony room. Water was freely available in group and holding cages. This regimen maintained rats at approximately 90 % of their free-feeding weights. The colony room was maintained on a 12:12-h dark/light cycle, and experimental sessions began within 3 h of the beginning of the dark cycle.

### Apparatus and materials

#### Foraging arena

**Fig. 1:**
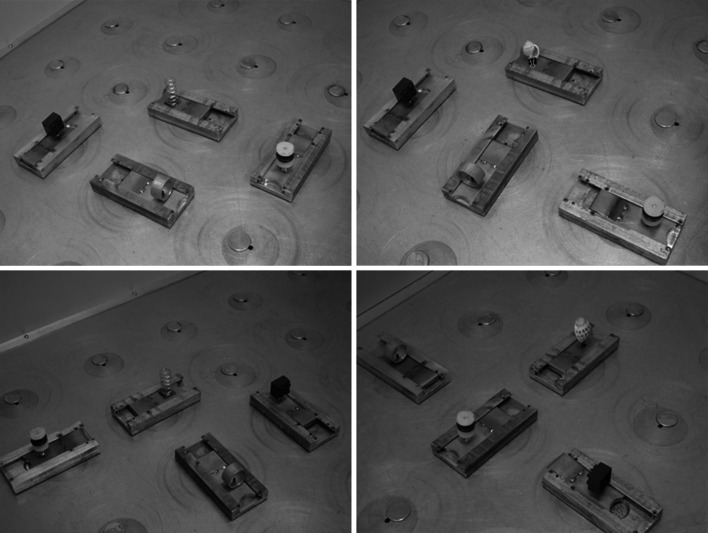
Five types of objects and the four different array *shapes* of oriented feeding stations shown in the foraging arena. Feeding stations are the type used in the first experiment (see text for description of modifications made to feeding stations in the second experiment)

This arena consisted of a 1.2-m square aluminum foraging platform that stood 56 cm above the floor of the experimental room. It was enclosed by 46-cm-high gray wood walls and surrounded by a black curtain suspended from the ceiling. In the first experiment, a 15-cm-high, 10-cm-wide black guillotine door occurred midway along each wall, but, as in Arain et al. (2012), only the guillotine door on north wall led to an attached standard stainless steel covered holding chamber and only it could be raised to allow rats to enter into and exit from the foraging arena. We note that the foraging arena contained no distinct directional cues to allow rats to determine which door would allow them to exit the foraging arena when they had completed a trial. Therefore, to allow animals in the second experiment to more easily find the operable door, we covered the three walls with the inoperable doors with distinctly different visually patterned poly-foam (Centra^®^) boards as follows. The wall opposite the one with the operable door was white, that to its right was dark blue, and that to its left had vertical dark blue and white 5-cm-wide stripes. We considered that these wall cues would serve as panoramic distal landmarks that, according to a recent model of animal navigation (Sheynikhovich et al. 2009), allow rodents to perceive their spatial orientation and the locations of proximal landmarks or beacons. A webcam (Logitech) was positioned 100 cm above the wall with the operable door. It was connected to a nearby laptop computer from which the experimenter monitored and recorded the rat’s search behavior. The floor of the arena contained twenty-five 2-cm-diameter holes arranged in a 5 × 5 matrix. As seen in Fig. 1, holes not covered by feeding stations were capped with aluminum disks. A 60-W incandescent lamp suspended 2 m above middle of the foraging area illuminated its interior.

#### Feeding stations, bait, objects

Although we used junk objects similar to those in Arain et al. (2012), we did not place them over uncapped recessed food wells on the floor of the arena. Rather as shown in Fig. 1, we placed them on metal plates that cover food wells on moveable raised rectangular feeding stations as in Arain and Cohen (2013). Each feeder as shown in Fig. 1 was a rectangular (16.5 cm × 7.6 cm × 2.5 cm) aluminum block containing a 2-cm-diameter, .5-cm recessed food well, covered by a 200-g stainless steel metal plate. A rat could uncover the food well by pushing the plate back with its nose. A vertical tube extending from the bottom of the feeder (not shown) allowed it to be positioned into an uncapped floor hole and oriented in any direction in the foraging arena. We note these feeders were too heavy for rats to change their experimenter-chosen orientations. In the first experiment, the rat could completely push each feeder’s cover plate completely off its food well. A feeder’s food well contained a perforated false floor under which was packed with inaccessible unsalted roasted sunflower seeds. A feeder was only considered baited if its food well also contained an accessible sunflower seed on top of its perforated floor. We modified these feeders for the second experiment by removing their perforated food well floors and inserting a removable and undetectable pin under each feeder that, when in place, prevented a rat from pushing its cover plate completely off the food well. This modification allowed us to bait each feeder’s food well with a sunflower seed that was only accessible when its feeder’s locking pin had been removed.

Although Fig. 1 shows five different types of junk objects on these feeders, each rat only received a set of four experimenter-selected objects. A flat-head metal screw was embedded into each object’s base to allow the experimenter to easily attach and remove any object from a magnet embedded into any feeder’s food well cover plate. There were three other replicates of each object to allow the experimenter to substitute one object for another between study and test arrays or place identical objects of any type on each feeder for a trial’s a test array. This figure shows four different asymmetrically polygon-shaped arrays derived from each rectangular feeder’s orientation. Thus, a feeder’s orientation provided its object with two spatial features, its feeder’s orientation relative to that of the other feeders in the array and the direction toward one of the foraging arena’s walls that a rat had to face when trying to move a feeder’s food well cover plate. We created differently shaped array’s feeders more similar to those in Arain and Cohen (2013) than to a square array of objects employed by Arain et al. (2012). Unlike any rats in Arain and Cohen (2013) that experienced all possible polygon-shaped arrays over trials, each rat in the present study was exposed to a differently shaped array that did not change throughout its experiment. We used such polygon-shaped arrays to insure that a rat would notice any change in its array's orientation from that of its study array that would occur on some post-acquisition (probe) trials to test if a rat had developed a representation of its array's shape. Obviously, a square-shaped array could only have a single orientation regardless of how we changed the positions of the objects.

## Procedures

Each experiment consisted of three phases: a feeder response shaping phase, a missing-object recognition training phase, and a missing-object recognition probe trial phase. The twelve animals in each experiment were randomly divided into two equal batches of six animals with three animals randomly assigned to each group in each batch. Therefore, each group contained a total of six animals that had to complete its experiment before the next six animals in the other batch for that experiment started it.

### Feeder response shaping phase

Prior to receiving missing-object recognition training and a post-training with occasional probe trials interspersed among regular baseline trials, rats were trained to enter the foraging arena and open four object-cued feeding stations, each baited with six seeds, rather than four other non-baited feeders without objects. These two types of feeders were randomly located and differently oriented in the arena. These shaping and preliminary training procedures closely followed those in previous studies from our laboratory (Cohen et al. 2010; Arain et al. 2012). However, rats in the first batch of six rats in the first experiment received one trial per day for 30 sessions, but the second batch in that experiment and all rats in the second experiment received two widely distributed (2 h) trials per day for 20 sessions before being transferred to the actual missing-object recognition task. We increased the number trials per day over fewer sessions for the remaining rats to match the pre-task training procedure used in earlier studies from our laboratory. We note that we changed this pre-training procedure in the first experiment after finding that the first batch of rats required considerably more trials (50–62 trials) to master the next phase’s missing-object recognition task than rats in the earlier studies from our laboratory. The second batch in this experiment and rats in the second experiment, however, required far fewer trials (14–20 trials) to acquire the missing-object recognition task, making their acquisition comparable to that of rats in the previous research. Following this initial training phase, we randomly and equally divided each batch of rats into the Fixed Configuration and the Varied Configuration group.

### Missing-object recognition training phase

We first describe the basic procedures used in this and the third phase of the first experiment and then our modifications of them in the second experiment.

Each rat within its assigned group was exposed to only one of the four arrays whose shape was determined by its differently oriented feeders as shown in Fig. 1. Each rat was also randomly assigned to any one of five possible sets of four objects also shown in Fig. 1. Every rat in the Fixed Configuration group received each of its four objects always on its specifically assigned oriented feeder within the array so that no rat had the same fixed configuration of objects. Every rat in the Varied Configuration group received each of its four objects on different oriented feeders over trials without having the same within-array configuration of objects occurring between consecutive trials. The location of a trial’s study and test array occurred in a fixed location that differed for each rat in the Fixed Configuration group. For rats in the Varied Configuration group, the location of its study and test arrays also did not vary within a trial but was changed to completely different non-overlapping locations between trials. In either group, an array was never located directly in front of the foraging arena’s start/exit door.

A rat received two widely distributed segmented trials per day. A trial began with a study array consisting of three randomly selected object-cued feeders, each baited with one seed and a non-baited (empty) feeder without an object. After a rat had obtained all seeds from its study array, it was allowed to exit the foraging arena into the external smaller holding cage from which it was removed and placed into a separate, covered holding chamber beneath the foraging arena while the experimenter prepared the foraging arena for that trial’s four-object test array. During this time, the experimenter misted the arena with a lemon-scented detergent solution, dried this area, replaced the four feeding stations with four other stations arranged in the same pattern, and replaced the study array’s three objects with replicates and added the fourth previously missing object onto the feeder that replaced the study array’s objectless feeder. These complex test array preparations served to eliminate possible subject-induced olfactory cues left on the study array feeders and on objects that could have helped animals find a missing object. The experimenter required about 2 min to make such preparations before placing the rat back into the start/exit chamber for its trial’s missing-object recognition test. Thus, each trial had a 2-min inter-array interval. After finishing its first trial of a session, the rat had to wait approximately 2 h in an individual holding cage outside the experimental room before being run on its second trial of the day.

The major procedural difference in the second experiment was that we randomly rotated the study and test arrays 90° or 180° or 270° over but not within trials for each group. The point of rotation was near the center of the training array, subject to the constraints imposed by the fixed grid on which the feeders were placed. The Varied Configuration group’s rats also had their arrays also moved to different locations over training trials, while the Fixed Configuration group’s rats had their arrays kept in the same location. A rat was transferred to the next phase after it found the test array’s ‘target’ object on its first choice on nine out of its last twelve trials (75 % criterion) or until a rat in the first batch of the first experiment had completed 60 trials (30 sessions) and any of the remaining rats in either experiment had completed 20 trials (10 sessions), which ever came first. This difference in when a rat might be transferred to the final phase reflects the fact that for some reason in the first experiment, only two animals in the first batch reached the 75 % criterion by their 54th trial, while five animals in the second batch did so within their first 20 trials. Those animals that did not reach this criterion by their final block of 12 trials, however, did find the missing object on their first choice on more trials than would be expected by chance.

### Post-training probe trial phase

**Fig. 2:**
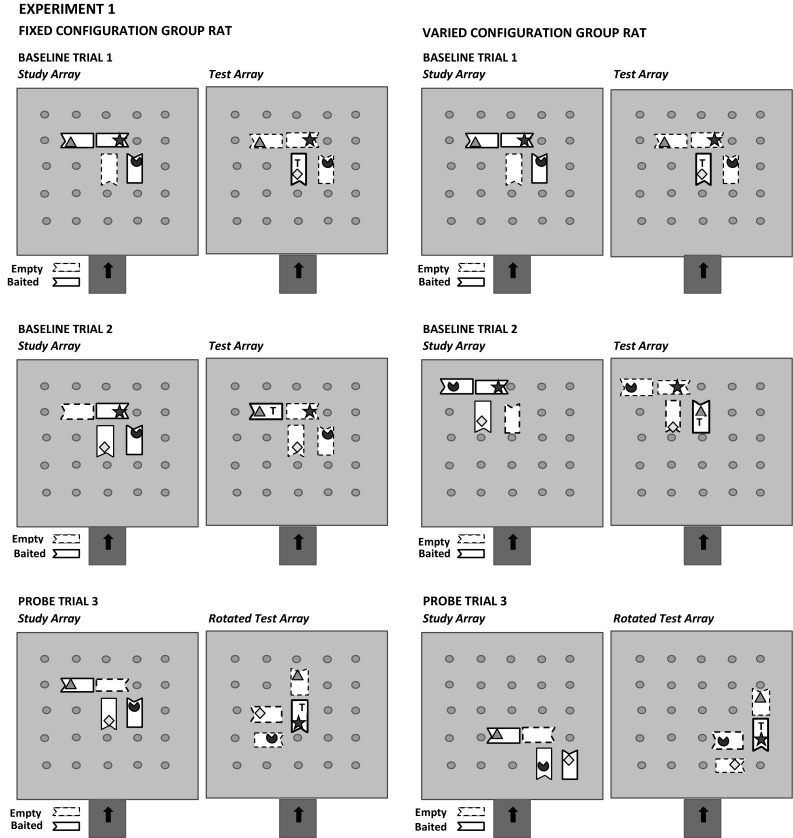
Examples of two training or baseline trials and a probe trial with their respective three-object study and four-object test arrays for an illustrative rat in each group in the first experiment. The target (*T*) icon in the test array represents the missing target object from that trial’s study array. In each group’s rats, only the probe trial’s test array is rotated from the orientation of its study array. The relative position of each object (icon) within each trial’s study and test array is fixed for a Fixed Configuration group rat and varied over trials but not within any trial for a Varied Configuration group rat. The location of a trial’s study and test arrays only changed over trials in the Varied Configuration group’s rat. Any feeder depicted by an *unbroken border* is baited, but any feeder depicted by a *dashed border* is un-baited. The *indented* portion of the *rectangular feeder* represents the front of the food well cover from where the rat had to push to uncover its food well as shown in Fig. 1. The sequence of the two training and a probe trial does not represent the actual sequence of a baseline and probe trials within a two-trial session nor are examples of a probe trial’s two other test array rotations shown in this figure (see text for further description)

In the first experiment, rats received 12 probe trials interspersed among 36 regular training trials (18 sessions). A session with a probe trial always had a regular training (baseline) trial that occurred either 2 h before or after the probe trial equally often. A probe trial’s test array was rotated either 90°, 180°, or 270° equally often in a random sequence without the same rotation being repeated on two probe trials in a row. The point of rotation was near the center of the training array, subject to the constraints imposed by the fixed grid on which the feeders were placed. The other, baseline trial in the session with a probe trial session did not have its test array rotated. An example of these two types of trials for a possible rat in each group in the first experiment is shown in Fig. 2.

**Fig. 3:**
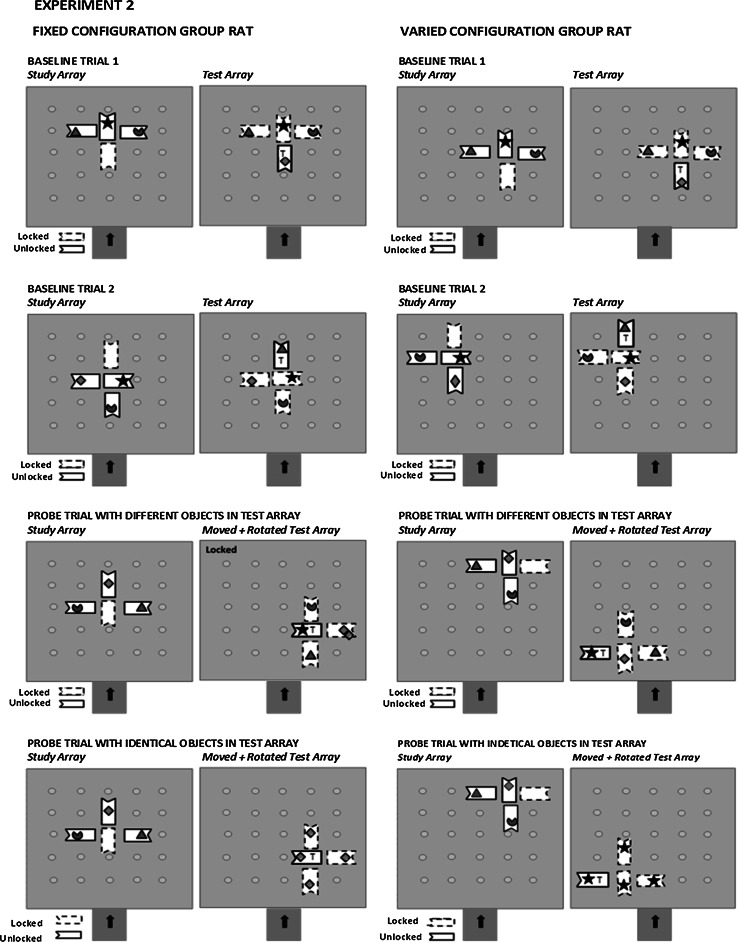
Examples of two training or baseline trials and two types of probe trials with their respective three-object study and four-object test arrays for an illustrative rat in each group in the second experiment. The target (*T*) icon in the test array represents the missing target object from a training/baseline trial and the first probe trial’s study array, but in the second probe trial, it represents a previously objectless target feeder in a test array with identical objects that may be either replicates of the missing object or of one of the three previously presented objects (see text for further details). The relative position of each object (icon) within each trial’s study and test array is fixed for a Fixed Configuration group rat and varied over but not within trials for a Varied Configuration group rat. The orientation of each array of feeders is rotated over but not within training/baseline trials for a rat in the Fixed Configuration group and in the Varied Configuration group. The array locations also change over trials but not within trials only for the Varied Configuration group’s rat. Any feeder depicted by an *unbroken border* has its food well cover unlocked, but any feeder depicted by a *dashed border* has its food well cover locked. The *indented* portion of the *rectangular feeder* represents the front of the food well cover from where the rat had to push to uncover its food well as shown in Fig. 1. The sequence of the two training and a probe trial does not represent the actual sequence of a baseline and probe trial within a two-trial session nor are the all three types of probe trial test array changes shown in this figure (see text for further details)

Rats in the second experiment received 24 probe trials interspersed among 48 regular training trials. The first 18 probe trials contained test arrays with four different objects, while the last six probe trials contained test arrays with identical objects that were replicates of the missing object on three of these probe trials’ test arrays and replicates of any one of each of the other three probe trial’s study array objects. Within each set of probe trials, that is, the first 18 probe trials with test arrays of different objects and the last six probe test trials with identical test array objects, one-third (six probe trials in the first set and two probe trials in the second set) had their test arrays rotated as in Experiment 1 (rotated probe trial test arrays), another third had their test array locations changed from those of their respective study arrays but not rotated (moved probe trial test arrays), and the remaining third had their test arrays both rotated and moved (rotated + moved probe trial test arrays). Each type of probe trial occurred randomly without repetition over consecutive trials. Figure 3 depicts an example of two training/baseline trials and a probe trial with a test array of different and one with identical objects for a possible rat in each group in the second experiment.

### Dependent measures and statistical analyses

We monitored the number of choices each rat made to find a missing object in each trial’s test array during each experiment’s second and third phase. We measured a rat’s missing-object recognition accuracy during the training phase by calculating the proportion of trials it found a missing object on its first choice or by its second choice over its first and last twelve trials in each experiment. To determine the effects of rotating probe trials’ test arrays in the first experiment, we calculated the proportion of the twelve probe and their accompanying baseline trials a rat found a missing object on its first and by its second choice. In the second experiment, we also calculated the proportion of the six probe and their accompanying baseline trials a rat found the missing object on its first and by its second choice when each set of probe trials’ test array with different objects had been rotated, moved, or both rotated and moved. We note there were too few of each type of probe trial with test arrays of identical missing or study array object replicates to reliably calculate similar proportions. Therefore, we calculated the mean number of choices each group’s rats made to find a test array’s baited feeder on each type of probe trial’s test array and on its accompanied baseline trials. The results of our statistical analysis of these data are presented in a supplementary section of this report. These results allowed us exclude each rat’s two probe trials with moved but not rotated test arrays because rats were not affected by this manipulation. Either rotating or rotating and moving a prove trial’s test array similarly affected rats test array performance; therefore; we combined the two types of probe trials into a block of four probe trials with rotated test arrays. We were then able to calculate the proportion of these four trials each rat found the target feeder on its first and by its second choice. We also excluded data from the six probe trials with moved test arrays and their accompanied baseline trials from among the first set of 18 probe trials and their baseline trials and combined six probe trials with rotated test arrays with the six probe trials with rotated and moved test arrays and their respective baseline trials for the same reasons as described in the results section of the main report. We partitioned the remaining 12 probe trials and their accompanying baseline trials into three successive blocks of four trials each from which we calculated the proportion of trials a rat found a missing object on its first and by its second choice within each block.

We analyzed these missing object or target feeder recognition data from each experiment by appropriate multi-factor ANOVAs to determine reliability of observed differences between and within groups. We also conducted one-sample, one-tailed *t* tests for each group to determine whether the proportion of trials it found a test array’s only baited feeder on its first or by its second choice was significantly greater than would be expected (first choice: .25; by second choice: .50). Effects were considered significant at *p* < .05. Although we report significant *F* values that confirmed observed functions involving group effects, we only indicate whether a group’s performance was significantly above chance when a *t* (*df* = 5) >2.014. Unless otherwise noted, the first and second *F* values reported for any significant effect are for the strict (on first choice) and lax (by second choice) test array performance measures in that order.

## Results

### Missing-object recognition training phase

Although all rats were able to find the missing object on their first choice over their last twelve trials above chance in each experiment, rats in the Varied Configuration group were less likely do so on nine of those trials, our acquisition criterion. Only one animal in the first experiment and three animals in the second experiment in their respective Variable Configuration group were able to reach this 75 % criterion. Within each Fixed Configuration group in these two experiments, however, only one rat in the first batch of the first experiment failed to reach this criterion. Thus, the proportion of all Fixed Configuration Group rats (11/12) that reached criterion was significantly greater than that (4/12) of all Varied Configuration group rats (Fisher test: *p* = .01).

**Fig. 4:**
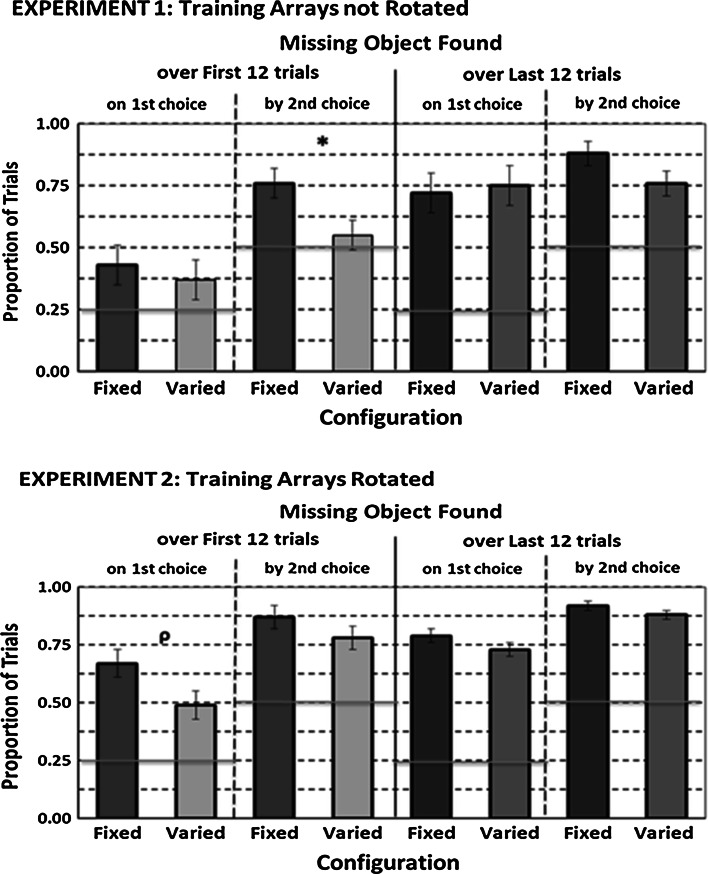
Mean proportion of trials each configuration group found a previously missing object on a first choice and by a second choice on its first 12 training trials (*left panel*) and last 12 training trials (*right panel*) in the first experiment (*upper graph*) and second experiment (*lower graph*). The *vertical error bars* represent ±SEM, and the *horizontal line* in each graph represents chance performance. Group data summary *bars with heavy borders* are for data significantly (*p* < .05) above chance performance. *Symbols* above a pair of Fixed and Varied Configuration *group bars* reflect a difference at or below the following probabilities: *p < .05; *ρ* = .065

Figure 4 shows the proportion of trials in the first and last block of twelve training that rats in each group found a missing object on their first and by their second choice. As seen in this figure’s graphs for the first and second experiments, the Fixed Configuration group appeared to find a missing object more accurately than the Varied Configuration group within the first block of trials. Although this difference was only significant in the first training block for the lax measure of missing-object recognition in the first experiment, *F*_1,10_ = 6.72, *p* = .027, only the Fixed Configuration group found a missing object significantly above chance for either measure. Differences between groups in the first block in the second experiment barely missed significance for the strict measure, *F*_1,10_ = 4.66, *p* = .056. In the last training block in either experiment, both groups developed missing-object recognition accuracy levels significantly above chance that were not significantly different from each other. The fact that more rats in the Fixed Configuration group than in the Varied Configuration group achieved criterion for finding the missing object on their first choice supports the idea that the former had less information to process about a missing object than the latter.

### Post-training probe trials phase

**Fig. 5:**
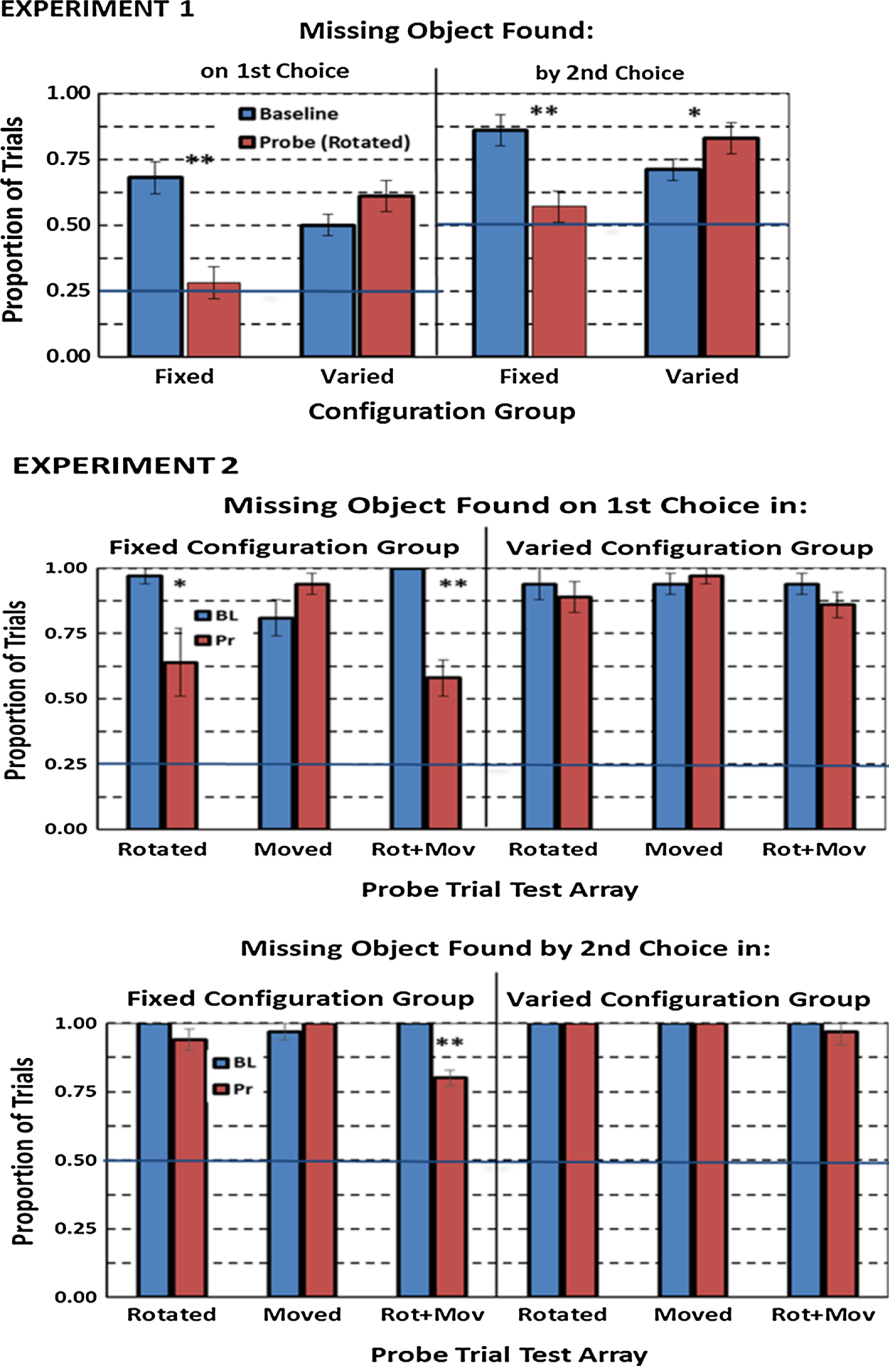
Mean proportion of trials each configuration group found a missing object on a probe (Pr) trial test array and its accompanying baseline (BL) trial test array on a first choice and by a second choice in the first experiment (*upper graph*) and in the second experiment (*middle* and *bottom graphs*). The *vertical error bars* represent ±SEM, and the *horizontal line* in each graph represents chance performance. Group data summary *bars with heavy borders* are for data significantly (*p* < .05) above chance performance. *Symbols* above any pair of Fixed and Varied Configuration *group bars* reflect a difference at or below the following probabilities: *p < .05; **≤.01

Figure 5 shows the proportion of trials that rats in each group found a missing object on their first and by their second choice on probe trials with test arrays consisting of different objects and on their accompanied baseline trials in each experiment. The graph for the first experiment shows that rotating probe trials’ test arrays reduced the Fixed Configuration group’s missing-object accuracy to chance and from baseline levels but slightly increased it in the Varied Configuration group for both measures of accuracy. These observations were supported by a significant Group by Trial Type (baseline vs. probe) interaction, *F*s_1,10_ = 49.37; 45.12, *p*s < .001, resulting from a significant decline in both measures in the Fixed Configuration group, *F*s_1,5_ = 100.84; 39.33, *p*s < .01, and from a significantly increased accuracy for the lax measure in the Varied Configuration group, *F*_1,5_ = 9.27, *p* = .027. Although a similar enhancement effect in the latter group for the stricter accuracy measure was not significant, *F*_1, 5_ = 3.51, *p* = .12, we note that four of its six animals improved their performance, while one decreased it and the other showed no change.

The graphs for the second experiment in this figure show that rotating or both rotating and moving a probe trial’s test array similarly reduced the Fixed Configuration group’s missing-object recognition accuracy but did not affect the Varied Configuration group’s missing-object recognition. Moving a probe trial’s test array without rotating it did not affect either group’s missing-object recognition. These observations were supported by a significant Group by Trial Type (probe; baseline) by Probe Trial Test Array type (rotated, moved, rotated + moved) interaction, *F*s_2,20_ = 3.83; 8.51, *p*s = .039; .002. This triple interaction resulted from a significant Trial Type by Probe Trial Test Array interaction only within the Fixed Configuration group, *F*s_2,10_ = 7.07; 14.73, *p*s ≤ .012. This double interaction resulted from that group significantly reducing its accuracy for finding a missing object on its first choice when a probe trial’s test array had been rotated or both rotated and moved, *F*s_1,5_ = 6.65; 33.94, *p*s = .05; .002, and by its second choice when a probe test array had been both rotated and moved, *F*_1,5_ = 54.39, *p* = .001. Failure to replicate the enhancement effect in the second experiment’s Varied Configuration group seems related to a higher baseline performance in this experiment than in the first experiment. As seen in Fig. 5 and confirmed by significant effects for each accuracy measure, *F*s_1,10_ ≥ 523.68, *p*s ≤ .001, each group’s performance on baseline trials that accompanied probe trials in the second experiment was practically perfect and considerably higher than that for its corresponding group in the first experiment. Therefore, a ceiling effect of high baseline performance in the second experiment could have simply obscured any possible probe trial enhancement in its Varied Configuration group.

**Fig. 6:**
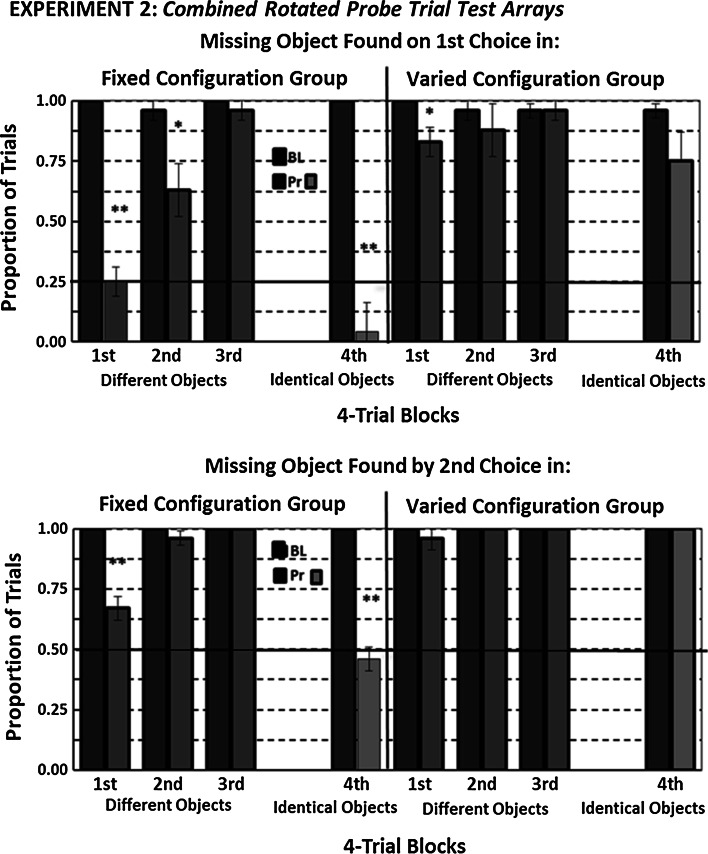
Mean proportion of trials each configuration group in the second experiment found a probe trial’s rotated test array’s missing object (Pr—*red bars*) or in a test array with identical objects (Pr—*orange bars*) and on each of its accompanying baseline (BL) trial test array on the first three blocks and the fourth block of trials, respectively, on a first choice (*upper graph*) and by a second choice (*lower graph*). The *vertical error bars* represent ±SEM, and the *horizontal line* in each graph represents chance performance. Group data summary *bars with heavy borders* are for data significantly (*p* < .05) above chance performance. *Symbols* above any pair of Fixed and Varied Configuration group bars reflect a difference at or below the following probabilities: *p < .05; **≤ .0 1

Figure 6 shows the proportion of trials each group in the second experiment found the remaining baited feeder on its first or by its second choice within each successive block of four probe trials with rotated test arrays (collapsed over location manipulation) where the last block’s probe trials test arrays contained identical objects. Data from probe trials with only moved test arrays and their accompanied baseline trials are not included in this figure for reasons we have already discussed. We note, however, that on the last two probe trials with moved, non-rotated test arrays with identical objects, nine out of twelve animals found the remaining baited feeder on their first choice on both these probe trial’s test array, while the other three rats, all in the Fixed Configuration group, found it on their first choice on one trial and by their second choice on the other trial.

We conducted two separate comparisons between groups from data shown in Fig. 6. First, we looked for any changes in probe trial performance over each group’s first three blocks. Then, we determined whether removing objects’ non-spatial information from probe trials’ test arrays in the final block affected rats’ accuracy for finding the remaining baited feeder compared to their ability to find it on the third block when such information was available.

As seen in the first three blocks of probe trials, the Fixed Configuration group reduced its accuracy for finding a missing object to chance on its first choice and to a lesser degree by its second choice on the first block but increased its accuracy up to baseline levels over the next two blocks. The Varied Configuration group showed only a slight decline in finding a missing object on its first choice in the first block of probe trials. These observations were confirmed by a significant Groups by Trial Type (probe vs. baseline) by Blocks interaction for each measure, *F*s_2,20_ = 6.52; 10.75, *p*s = .007; .001, resulting from a significant interaction between the Blocks and Trial Type only within the Fixed Configuration group, *F*s_2,10_ = 17.38; 19.00, *p*s ≤ .001. We also conducted a similar comparison among three probe trial blocks in the first experiment without uncovering any amelioration in the adverse effects from rotating probe trials’ test arrays in the Fixed Configuration group. Thus, the somewhat overall lesser disruptive effect from rotating probe trials’ test arrays seen in Fig. 5 in the second experiment’s Fixed Configuration group was not evenly distributed over all probe trials but resulted from an initial substantial but transient adverse effect.

Comparisons between the last two blocks revealed that only the Fixed Configuration group reduced its probe trial test array accuracy on its fourth block to below chance on its first choice and to chance by its second choice. The Varied Configuration group only slightly but nonsignificantly reduced its accuracy on its first choice. These observations were confirmed by a significant interaction among all three factors for each accuracy measure, *F*s_1,10_ = 14.90; 49.71, *p*s ≤ .003, resulting from an interaction between the two within-subject factors only in the Fixed Configuration group, *F*s_1,10_ = 302.50; 49.71, *p*s ≤ .001. Thus, removal of non-spatial information of a missing object only prevented the Fixed Configuration group from using its missing object’s relevant spatial information from the study array, that is, its feeder’s orientation in a rotated test array.

## Discussion

The main question in this study is how well rats’ visuospatial working memory in a foraging task corresponds to that observed in humans tested in non-foraging tasks. Specifically, we tested whether rats’ visuospatial working memory conforms to the limited capacity hypothesis observed in human working memory (see Cowan 2002, 2008 for reviews of this notion). In our preparation, we tested the prediction derived from this hypothesis that the amount of information about each object encoded into and retrieved from rats’ visuospatial working memory will inversely affect their missing-object recognition accuracy. We manipulated the amount of spatial and non-spatial information about each object a rat would separately process during training by varying the consistency of pairing these features within each object over trials. As expected, rats in the Fixed Configuration groups acquired the missing-object recognition task more easily than those in the Varied Configuration group presumably because the former had no or less separate non-spatial and spatial information to process and retain about each object. Results from occasional post-acquisition rotations of a trial’s test array provided a more definitive test of the limited capacity hypothesis. In the first experiment, this manipulation reduced missing-object recognition accuracy to chance in the Fixed Configuration group, while it slightly but reliably enhanced it in the Varied Configuration group. These differences were attributed to the Varied Configuration group’s rats being able to reduce the load of information they needed to retrieve to find a missing object to only relevant non-spatial information on such probe trials. The Fixed Configuration group’s rats could not do this as the information they could only retrieve about a missing object consisted of that object’s fully integrated non-spatial and spatial features.

The prediction that rotating arrays over training trials in the second experiment would prevent its Fixed Configuration group from being disrupted from occasional within-trial test array rotations was not supported. Rather this group displayed initial severe disruption that disappeared but then re-emerged when its probe trials’ rotated test arrays consisted of identical objects. Instead of forming only a single representation of each object’s non-spatial features integrated with its feeder’s fixed relative orientation, these rats could have also developed a separate representation of each object’s trial-specific feeder direction. Perhaps their initial disruption from probe trial rotations might have reflected their initial attempt to use a missing object’s uninformative trial-specific feeder direction. Their recovery from this disruption could indicate that they stopped retrieving such useless spatial information and only maintained or switched to retrieving a representation of the missing object’s relevant integrated spatial and non-spatial features. Thus, this group’s rats appeared to use a flexible retrieval strategy by eventually relinquishing their retrieval of a missing object’s trial-specific feeder direction during rotated probe trial test arrays. That neither group in the second experiment was adversely affected when a probe trial’s test array was moved but not rotated in the absence of relevant non-spatial information suggests that all rats could use information only from a missing object’s relevant trial-specific feeder direction. We attributed a failure to find an enhanced probe trial effect in the second experiment’s Varied Configuration group to a general obscuring ceiling effect. The question remains what if any factors might have been responsible for the second experiment’s animals developing such superior baseline performance. A discussion of possible answers to this question follows after we consider how the difference in probe trial effects between each experiment’s Fixed Configuration groups corresponds to human matching accuracy of natural multi-featured objects.

The persistent adverse effects from probe trial test array rotations in the first but not in the second experiment’s Fixed Configuration group may correspond to the natural complex object-inversion effect found in humans and other primates. As previously discussed, only after having had considerable experience with objects in their normal (more upright) orientations do human and non-human primates show the disruptive inversion effects presumably because they no longer separately process each object’s internal features (Diamond and Carey 1986; Parr et al. 1998; Gauthier et al. 2000; Parr and Heintz 2006, 2008; Curby et al. 2009). In our preparation, rats may have also perceived their assigned arrays of differently oriented adjacent feeders as a complex polygon-shaped object when its internal features (junk objects) occurred in fixed positions for some rats (the Fixed Configuration groups). Only in the first experiment, however, did each rat in the Fixed Configuration receive its assigned complex object-like array in a fixed (non-rotated) orientation on training/baseline trials. Thus, when confronted with the same occasionally rotated test array, a rat may not have perceived it as otherwise identical to its study array just as ‘experts’ have difficulty in recognizing a complex rotated object is the same as an upright one. This account extends the ability of rats to represent and use a geometrical module of rectangular arrays of adjacent objects within a larger area (Gibson et al. 2007) to more complex asymmetrically shaped polygons. Support for an extension of the geometrical module model requires that rats be similarly affected by transformations of such polygon-shaped arrays as they have been with rectangular arrays of adjacent objects (Gibson et al. 2007) or in larger enclosed rectangular spaces (Cheng and Gallistel 2005). We approach this idea with some caution, however, as it assumes that a rat in the Fixed Configuration group could have somehow developed a perceptual representation of the fixed orientation of its array in the absence of obvious distal directional cues within the foraging arena in the first experiment. Perhaps those rats could have noticed and used any of the hallway and the running room stimuli or kept track of direction using interim inertial cues while they were being transported to and from the foraging arena between trials.

Provided rats can be prevented from perceiving directional extra-foraging arena cues, a test of the array-inversion hypothesis requires co-variation of array orientation stability and the amount or distinctiveness of directional foraging arena wall cues over trials during initial training. In the absence of such a design, we must also consider other factors related to rats’ foraging behavior that could produce only analogous similarities between human and rat visuospatial working memory. We discuss two possible foraging-specific factors: the type of learning rats might have used to remember the location of a target (objectless) feeder in a study array and the information they need to find their way home in a potentially dangerous open area even when foraging at short distances from ‘home’ (Etienne et al. 1996).

Skinner et al. (2003) found that rats have greater difficulty using place than either egocentric response (turn left or right) or allocentric direction information (go east or west) to find food at a fixed location in an open T-maze or field. However, in that study, rats could be trained to as easily use place information by being forced to begin searching from different starting positions. Later research from that laboratory (Stringer et al. 2005) revealed that hippocampal damage prevents rats from learning to use either directional or place information as easily as response information even under optimal training conditions. Given that rats also process these three types of information in working memory, our rats might have been more likely to approach arrays that were always rotated over trials in the second experiment from relatively different starting points. In the first experiment, they may have been more likely to approach arrays from the same starting point because they were exposed to unchanged array orientations. Consequently, the second experiment’s rats would have more easily learned to use within-array (relative) place information to remember the location of a study array’s objectless feeder than rats in the first experiment. The first experiment’s rats would have had to rely more on response and directional information that became irrelevant on probe trial rotated test arrays. Thus, the Fixed Configuration group’s rats would be adversely affected by probe trial test array rotations by only using its missing-object feeder’s incorrect trial-general directional or response information. But the Varied Configuration group’s rats in that experiment might have enhanced their missing-object recognition by dissociating the missing object from its trial-specific incorrect directional or response information. In the second experiment, both groups would have been able to use a missing object’s place information to generate better baseline performance in both groups and reduce differences between them on probe trial test array rotations.

Concerning the second type of information rats use to exit the foraging arena, rats in the first experiment like those in Arain et al. (2012) had no discernable directional distal landmark (wall) cues to help them find the operable exit door. Rats in the second experiment had such wall cues. Therefore, the first experiment’s rats would have had to construct egocentric path integration information perhaps in the form of vector sum representations (Cheng 1989, 1994; Etienne et al. 1998) from a study array’s objectless feeder and retain this information while they searched for the missing object in a trial’s test array. Rats in the Varied Configuration group would have had to continuously update such vector sum information on each trial from ever moving arrays over trials and could only retain such varied information as trial-specific working memories. As rats in the Fixed Configuration group experienced their arrays in the same location over trials, they might only have formed a few stable vector sum representations associated with each object-cued feeder to be stored in and activate from reference memory while searching for the missing object. Rats in the Varied Configuration group might have had more difficulty in finding the missing object in training/baseline trials because they had more varied and a greater amount of path-integrated information to actively maintain during their test array searches than rats in the Fixed Configuration group. But on probe trial rotated test arrays, the Varied Configuration group rats might be able to more easily dispense with retrieving such burdensome, uninformative trial-specific navigational information than could Fixed Configuration group’s rats. Consequently, the Varied Configuration group’s rats would enhance their missing-object recognition performance by reducing the amount of spatial information they normally use on baseline trials. The Fixed Configuration group’s rats would be severely disrupted on such probe trial test arrays by continuing to use uninformative navigational information activated from their reference memory.

In the second experiment, however, rats could use simpler and thus easier-to-remember directional wall cues in place of or as primary information to find the operable exit door after having found the missing object. Thus, animals in this experiment may have developed better test array performance because they had less homebound information to actively maintain than animals in the first experiment while searching for a missing object.

The transient disruption by the second experiment’s Fixed Configuration group on probe trials’ rotated test arrays could reflect that these rats also formed and used integrated homebound paths as secondary backup information. This possibility is in accordance with research, showing that rodents will form and use path-integrated analyses even when well-defined landscapes provide sufficient directional information (Wishaw and Tomie 1997; Etienne and Jeffery 2004; Etienne et al. 1996; Vlasak 2006). With only a few directional landmarks in otherwise barren environments, however, such animals rely primarily on path integration and use external cues as secondary corrective references (Benhamou and Poucet 1996; Benhamou 1998). That the Varied Configuration group’s rats never displayed any such transient probe trial effects suggests that they relied solely on fixed wall cues after completing their search in the test array. Furthermore, these animals would have had to construct many more different trial-specific integrated homebound paths for each oriented feeder by having had their arrays both rotated and moved over training and baseline trials than rats in the Fixed Configuration group that only had their arrays rotated over trials. A more formal Bayesian analysis (Cheng et al. 2007) would clearly predict that the Varied Configuration group should more strongly prefer using directional wall cues over navigational path information than the Fixed Configuration group.

## Electronic supplementary material

Supplementary material 1 (DOCX 21 kb)

Supplementary material 2 (DOCX 14 kb)
