# Supplementary material for: Rats (Rattus norvegicus) flexibly retrieve objects’ non-spatial and spatial information from their visuospatial working memory: effects of integrated and separate processing of these features in a missing-object recognition task
Source: Anim Cogn. 2015 Aug 27;19:91–107. doi: 10.1007/s10071-015-0915-8 (PMC4701772; doi:10.1007/s10071-015-0915-8)
Supplement: Supplementary file 1 — Supplementary material 1 (DOCX 21 kb) [file 10071_2015_915_MOESM1_ESM.docx]

|  |  |  |  |  |  |  |  |  |
| --- | --- | --- | --- | --- | --- | --- | --- | --- |
|  |  | | | | | | | |
|  |  |  |  |  |  |  |  |  |
|  |  |  |  |  |  |  |  |  |
|  |  |  |  |  |  |  |  |  |
|  |  |  |  |  |  |  |  |  |
|  |  |  |  |  |  |  |  |  |
|  |  |  |  |  |  |  |  |  |
|  |  |  |  |  |  |  |  |  |
|  |  |  |  |  |  |  |  |  |
|  |  |  |  |  |  |  |  |  |
|  |  |  |  |  |  |  |  |  |
|  |  |  |  |  |  |  |  |  |
|  |  |  |  |  |  |  |  |  |
|  |  |  |  |  |  |  |  |  |
|  |  |  |  |  |  |  |  |  |
|  |  |  |  |  |  |  |  |  |

**Supplementary Fig**. Mean number of choices to find the target (unlocked) feeder in a probe trial’s test array with identical objects in the second experiment. The vertical error bars represent + SEM and the horizontal line in each graph represents chance performance. A group’s data summary bar with a continuous border indicates that it was either perfect or not significantly different from perfect performance for finding the target feeder on the first choice. A group’s data summary bar with broken borders indicates its data was not significantly different from chance performance. Symbol ** over a pair test data bars indicates a significant difference between groups at *p <* .01. See Supplementary Results section for further description of statistical findings.
