# Supplementary material for: Rats (Rattus norvegicus) flexibly retrieve objects’ non-spatial and spatial information from their visuospatial working memory: effects of integrated and separate processing of these features in a missing-object recognition task
Source: Anim Cogn. 2015 Aug 27;19:91–107. doi: 10.1007/s10071-015-0915-8 (PMC4701772; doi:10.1007/s10071-015-0915-8)
Supplement: Supplementary file 2 — Supplementary material 2 (DOCX 14 kb) [file 10071_2015_915_MOESM2_ESM.docx]

Supplementary Results

Our examination of the number of choices each rat made to find the missing object on each of the baseline trials that accompanied each of the last six probe trials in the second experiment revealed that all but two rats found it on their first choice. The two exceptions, both from the Varied Configuration group, found it by their second choice. Given such perfect or nearly perfect baseline trial performance in the Fixed and Varied Configuration groups respectively, we only statistically examined the number of choices each group of rats made to find the target feeder on the probe trials’ test arrays by a 2 (group) by 2 (type of identical object replicates: study array; missing object) by 3 (type of probe trial test array: rotated; moved; rotated + moved) ANOVA with repeated measures on the last two factors. This analysis revealed significant effects for groups, *F* _1, 10_ = 2280.17, *p* < .001, type of probe trial test array, *F* _2, 20_ = 16.36, *p* < .001, and an interaction between these two factors, *F* _2, 10_ = 16.04, *p* = .001. Separate 2-way ANOVAs for each group revealed only a significant effect for type of probe trial test array within the Fixed Configuration group, *F* _2, 10_ = 16.04, *p* = .001. Pairwise comparisons revealed that this group made significantly more choices to find a target feeder on probe trials’ rotated or rotated + moved test arrays than on probe trials’ moved test arrays. No significant difference was found between the two types of probe trial rotated test array performance in this group. Given that type of identical test array object had no observable or statistical effects, we summarized these findings in supplementary figure collapsed over this factor. As seen in this figure and supported by pair-wise comparisons and subsidiary one sample t-tests, the Varied Configuration group showed practically the same near perfect test array performance for each type of probe trial while the Fixed Configuration group also showed a similar effect on moved probe trials’ test arrays. The Fixed Configuration group’s performance on probe trail rotated or on probe trial rotated + moved test arrays did not significantly depart from random choices. These findings supported our decision to combine both these types of probe trials and exclude those with only moved test arrays for the analysis of proportion of trials each group found the target feeder on its first or by its second choice as described in the main results section of this report.

[Supplementary Figure about here]
